# Decision-making on water–nitrogen irrigation for *Isatis indigotica* in oasis based on the coupling of fuzzy evaluation model and particle swarm optimization algorithm

**DOI:** 10.3389/fpls.2026.1890055

**Published:** 2026-08-11

**Authors:** Zeyi Wang, Yucai Wang, Fuqiang Li, Xuan Li, Caixia Huang, Peng Zhou, Lian Lei

**Affiliations:** 1 College of Water Conservancy and Hydropower Engineering, Gansu Agricultural University, Lanzhou, China; 2 Yimin Irrigation Experimental Station, Hongshui River Management Office, Zhangye, China

**Keywords:** fuzzy evaluation, *Isatis indigotica*, particle swarm optimization algorithm, regression model, water-nitrogen regulation

## Abstract

To quantitatively assess the responses of the growth, yield, quality and resource utilization of *Isatis indigotica* to water-nitrogen regulation and determine the optimal water-nitrogen management plan for *Isatis indigotica*, an oasis medicinal plant, field-grown *Isatis indigotica* under drip irrigation with plastic film mulching was taken as the research object. Three water levels (low W1: 60%–70%*θ_f_*, medium W2: 70%–80%θf, high W3: 80% –90%θf) and three nitrogen application levels (low N1: 150 kg·ha^-1^, medium N2: 200 kg·ha^-1^, high N3: 250 kg·ha^-1^) were set, with no water control (W0) and no nitrogen application (N0) as the control. Based on two-year experimental data, a multi-level weighted fuzzy evaluation model was used to comprehensively evaluate the growth, yield, quality and resource utilization indicators. A regression model between water-nitrogen factors and evaluation indices was established, and the Particle Swarm Optimization (PSO) algorithm was coupled for optimization and solution. The evaluation indices of yield and growth indicators under the medium water and medium nitrogen (W2N2) treatment were the highest (with average values of 0.1173 and 0.1125); the evaluation indices of resource utilization indicators under the low water or medium water and low nitrogen treatments were relatively high (with average values of 0.1220 and 0.1224); and the evaluation indices of quality indicators under the low water treatment were relatively high (with an average value of 0.1025). The secondary fuzzy comprehensive evaluation showed that W2N2 was the optimal treatment. The interaction between water and nitrogen was significant, and the effects of both on comprehensive growth showed a trend of increasing first and then decreasing. The optimal water-nitrogen combination obtained by PSO was: water level of 75.10%–85.10%*θ_f_* and nitrogen application rate of 224.53 kg·ha^-1^. This study can provide a basis for the scientific management of water-fertilizer in the local field cultivation of *Isatis indigotica*.

## 1 Introduction

In China, the stock of natural wild Chinese herbal medicine (CHM) resources has been continuously declining. Affected by long - term unregulated mining and the degradation of the regional ecological pattern, wild medicinal plants are becoming increasingly scarce, and the imbalance between supply and demand in the CHM market has become more and more prominent (Zhou et al., 2022a; Tang et al., 2021). Compared with chemically synthesized drugs, CHM have mild medicinal properties and relatively high safety, and their application scenarios in the modern medical system are constantly expanding (Zeng et al., 2023; Zhao et al., 2022; Fan et al., 2018). With the acceleration of the industrialization process of traditional Chinese medicine, the market demand for genuine medicinal materials from all sectors of society has been continuously rising. Implementing standardized large-scale artificial cultivation has become an important measure to make up for the gap in wild resources and ensure stable industrial supply (Li et al., 2016, Li et al., 2015). Research shows that excessive extraction of wild medicinal plants can easily lead to vegetation damage and the decline of genetic diversity of germplasm resources, while standardized artificial cultivation can not only conserve resources but also stabilize the internal quality of medicinal materials (Chen et al., 2016; Zhang et al., 2025). However, against the backdrop of the rapid expansion of artificial cultivation scale, problems such as unreasonable input of water and fertilizer and low resource utilization efficiency are widespread in the production areas of bulk medicinal materials represented by *Isatis indigotica*. This not only increases production costs but may also pose ecological and environmental risks. Therefore, at this critical period of industrial transformation, conducting research on the optimized water-nitrogen management of *Isatis indigotica* is of great practical urgency for achieving the coordinated goals of high yield, high quality production and resource conservation.

*Isatis indigotica* Fort., belonging to the Brassicaceae family, is a biennial herbaceous medicinal plant. Its dried fleshy root is a traditional and major genuine medicinal material, also known as Banlangen. Clinically, it is commonly used to clear heat and detoxify, cool blood, and relieve a sore throat. It is a core raw material in the research and development of various antiviral Chinese patent medicines and clinical diagnosis and treatment (National Pharmacopoeia Committee, 2015). *Isatis indigotica* has a growth habit of being drought-tolerant, light-loving, and cold-resistant, with excellent ecological adaptability, making it highly suitable for the natural climate conditions in the arid and semi-arid regions of northwestern China (Nie, 2024). Minle County, Gansu Province, with its superior light and heat resources, temperature difference conditions, and soil endowment, is titled “the hometown of Banlangen in China”. As a core advantageous production area of *Isatis indigotica* in the country, the local planting scale and total output of *Isatis indigotica* rank among the top in the country. The CHM industry has become a dominant industry that helps local rural revitalization and farmers increase their income and become rich (Qian, 2025). At present, crop cultivation in this region is still mainly based on traditional extensive management. Farmers’ field water-fertilizer inputs are quite arbitrary, and problems such as unbalanced irrigation water volume and blind increase in chemical fertilizers are common. This not only causes serious depletion of regional agricultural water-soil resources but also easily induces agricultural ecological risks such as farmland non-point source pollution (Pan et al., 2023; Yan et al., 2020). At the same time, it may inhibit the normal growth and development of the roots of *Isatis indigotica*, resulting in large fluctuations in the content of effective components in the medicinal materials and low utilization efficiency of field water-fertilizer resources. These practical problems seriously hinder the development of the local *Isatis indigotica* industry towards high-quality, green, and sustainable directions.

In recent years, many scholars have carried out a large number of experimental explorations on the field water-fertilizer management of medicinal plants. Qin et al. (2015) found through a formula fertilization experiment that an imbalance in the ratio of nitrogen, phosphorus, and potassium can significantly block the transfer and accumulation of dry matter in *Isatis indigotica* plants, and excessive nitrogen application can also significantly reduce the content of the core medicinal active ingredients in the roots. Zhou et al. (2022b) carried out a water deficit experiment on *Isatis indigotica* based on the climatic characteristics of the Minle production area in the Hexi Corridor. The results showed that deficit irrigation can reduce water consumption by 4.44%–10.21%, and continuous mild water deficit (65%–75%*θ_f_*) in the middle growth stage can simultaneously increase yield, water use efficiency (WUE), and the content of effective components. Tang et al. (2019) further confirmed that appropriate moisture conditions (75% medium water content) combined with a high nitrogen level (25 mM) can obtain the maximum root dry weight and leaf dry weight of seedlings, while an inappropriate water-nitrogen combination will inhibit root development. Xu et al. (2025) clearly pointed out that the coupling of the synergistic effect of water-fertilizer and the cross-scale feedback mechanism is the key to achieving efficient utilization of agricultural water resources. Optimized water-fertilizer management can increase *WUE* by 10%–15% and yield by 3.5%–4.5%, while the extensive water-fertilizer management mode will significantly reduce the partial productivity of fertilizers, further exacerbating the shortage of agricultural production water in arid areas (Zhao et al., 2021). Therefore, quantitatively analyzing and evaluating the water-fertilizer regulation effects of *Isatis indigotica* and seeking reasonable water-fertilizer application amounts is not only an objective need but also an urgent situation.

However, current research on the cultivation of *Isatis indigotica* mostly focuses on directions such as suitable cultivation zoning, single-nutrient regulation, and physiological responses to water stress. Most studies seek the best treatment through single crop factors, such as yield, quality, or WUE. In addition, in view of the actual production conditions of genuine medicinal materials planting in the oasis of the arid region in Northwest China, there is still a lack of systematic research on the construction of a comprehensive evaluation model by integrating multi-dimensional evaluation indicators such as growth, yield, quality, and resource utilization, and the development of an optimized decision-making for the efficient water-fertilizer utilization of *Isatis indigotica*. Therefore, this study takes *Isatis indigotica* planted by direct seeding in the field as the research object. Based on relevant measured indicators, a two-level fuzzy evaluation model is constructed from the three dimensions of high-yield, high-quality, and efficient resource utilization. A regression model between water-nitrogen factors and the evaluation index is established, and a multi-objective optimization model of the particle swarm optimization (PSO) algorithm is coupled to determine the optimal water-nitrogen management scheme for *Isatis indigotica* in the oasis, aiming to provide a theoretical basis and practical reference for the scientific control of water-fertilizer integration of similar medicinal plants in the northwestern arid region.

## 2 Materials and methods

### 2.1 Description of the study site

This experiment was conducted from May to October in 2018 and 2019 at the Yimin Irrigation Experiment Station in the Hongshui River Irrigation District, Minle County, Gansu Province (38°39’ E, 100°43’ N, with an altitude of about 2000 m). The experimental area has a dry climate and insufficient water resources, belonging to a typical continental desert-steppe climate. The average annual temperature is approximately 6°C, the extreme maximum temperature is 37.8°C, and the extreme minimum temperature is -33.3°C. The average annual total rainfall is 183–285 mm, the average annual frost-free period is 136 days, and the annual sunshine hours are about 3000 h. The soil is light loam, fertile in texture, with a field capacity of 24.0% (mass water content), a soil bulk density of 1.46 g·cm^-3^, and a groundwater level of about 20 m, unaffected by salinization.

### 2.2 Experimental design

The *Isatis indigotica* seeds used in the experiment were plump, large-grained, and uniform, provided by the local CHM cooperative. According to the actual growth process of *Isatis indigotica* in the local area, its growth period was divided into four stages: seedling (30 days), vegetative growth (60days), fleshy root growth (40days), and fleshy root maturity (25days). A two-factor split-plot design was adopted, with water regulation as the main treatment and nitrogen application as the sub-treatment. There were 3 levels of water regulation (W) and 3 levels of nitrogen application (N), namely low water (W1, 60%–70%*θ_f_*, *θ_f_* is the field capacity), medium water (W2, 70%–80%*θ_f_*), high water (W3, 80%–90%*θ_f_*); low nitrogen (N1, 150 kg·ha^-1^), medium nitrogen (N2, 200 kg·ha^-1^), high nitrogen (N3, 250 kg·ha^-1^, the common nitrogen fertilizer application rate of local farmers), and the control CK with no irrigation (W0) and no nitrogen application (N0). There were a total of 10 treatments, with 3 replicates for each treatment, resulting in 30 plots. Each plot was 8 m long, 3.75 m wide, with an area of 30 m², and the effective planting area of the experiment was 900 m². To prevent cross−seepage of soil water among treatments, 60 cm plastic film barriers were installed between different irrigation treatments. The experimental design is shown in **Table 1**. The drip irrigation under plastic film method was used. During the growth period, the soil moisture was controlled in the 0–60 cm soil layer, and the actual soil water content was basically within the upper and lower limits of the experimental set soil moisture control range. Flat–land film–covered drip–irrigation cultivation was carried out, with a film width of 120 cm, a plant spacing of 8–10 cm, an average row spacing of 10–15 cm, and a planting density of 800,000 plants·ha^-1^. Two drip irrigation tapes were laid on one film, with a row spacing of 42.5 cm between the drip-irrigation tapes, a dripper spacing of 30 cm, and a dripper flow rate of 2.5 L·h^-1^. A control valve was installed in each plot of the same irrigation level to control the irrigation amount of the irrigation area at any time. The water meter and pressure gauge were located at the drip-irrigation hub, and the normal irrigation pressure was 0.1 MPa.

**Table 1:** Experimental scheme for water-nitrogen regulation of *Isatis indigotica*.

Treatments	Soil moisture and nitrogen application level	Seedling	Vegetative	Fleshy root growth	Fleshy root maturity	Nitrogen application level/kg·ha^-1^
T1	W1N1	60∼70	60∼70	60∼70	60∼70	150
T2	W1N2	60∼70	60∼70	60∼70	60∼70	200
T3	W1N3	60∼70	60∼70	60∼70	60∼70	250
T4	W2N1	70∼80	70∼80	70∼80	70∼80	150
T5	W2N2	70∼80	70∼80	70∼80	70∼80	200
T6	W2N3	70∼80	70∼80	70∼80	70∼80	250
T7	W3N1	80∼90	80∼90	80∼90	80∼90	150
T8	W3N2	80∼90	80∼90	80∼90	80∼90	200
T9	W3N3	80∼90	80∼90	80∼90	80∼90	250
CK	W0N0	0	0	0	0	0

In the table, the letters W and N represent the water regulation level and nitrogen application level, respectively. The numbers 0, 1, 2, and 3 represent no irrigation or no nitrogen application, low water or low nitrogen, medium water or medium nitrogen, and high water or high nitrogen, respectively. T1-T9 represent the corresponding treatment numbers respectively. CK is the control treatment, and the values on both sides of “~” represent the percentage of field capacity.

### 2.3 Measurements and calculations

#### 2.3.1 Growth indicators

At the end of the growth period of *Isatis indigotica* (155 days), 5 plants with basically the same growth trend were selected from each plot for sampling. The plant height was measured with a millimeter-scale ruler; Leaf area was measured using a leaf area meter and then converted to total leaf area per unit ground area (i.e., leaf area index, *LAI*); The number of leaves was recorded, the length of the main root was measured with a millimeter-scale ruler, and the diameter of the main root (at the upper 1/4 of the root) was measured with a vernier caliper. The underground part (Banlangen) of the sampled plants was deactivated at 105°C for 30 min and then dried at 80°C until the mass was constant. The dry weight was recorded, and the dry matter accumulation was calculated according to the number of sampled plants.

During each growth period of *Isatis indigotica*, on a typical sunny day, the net photosynthetic rate (*Pn*) of the third functional leaf of *Isatis indigotica* plants was measured from 09:00–11:00 using an LI–6400 photosynthesis instrument. The measurement was repeated 3 times in each plot. To reduce the influence of time error, the measurement of the same plot was completed within 10 min.

#### 2.3.2 Yield indicators

During the harvest, the yield of each plot was weighed and calculated separately, and finally converting it into the yield per hectare. The yield of each treatment was the average of 3 replicates.

#### 2.3.3 Quality indicators

The contents of indigo, indirubin, and epigoitrin were determined by high-performance liquid chromatography (LC–10ATVP), and the specific determination method is shown in reference (National Pharmacopoeia Committee, 2015). The polysaccharide in *Isatis indigotica* root was determined by the phenol-sulfuric acid method, and the specific determination method is shown in reference (Wang, 2006).

#### 2.3.4 Resource utilization indicators

The calculation formulas for water use efficiency (*WUE*, kg·ha^-1^·mm^-1^) and nitrogen fertilizer partial productivity (*NFPP*) are as follows (Equations 1, 2):

(1) WUE=EY/ET

(2) NFPP=EY/N

where *EY* represents the economic yield of *Isatis indigotica*, kg·ha^-1^; *ET* represents the water consumption, mm; *N* is the nitrogen application rate, kg·ha^-1^.

#### 2.3.5 Methods and steps of multi-level fuzzy comprehensive evaluation

In practical work, when evaluating a thing, multiple factors or indicators are often involved. At this time, it is required to make a comprehensive evaluation of the thing based on these multiple factors or indicators, rather than just evaluating the thing from the situation of one factor or one indicator. This is a comprehensive evaluation. Based on the characteristics of multiple factors and fuzziness in the evaluation process, the specific steps of this study are as follows (Fu, 2006):

1. Establishment of the first-level fuzzy comprehensive evaluation model

- Determine the single-factor evaluation matrix.
- Determine the weight set of the subfactors. The subjective weight (*SW*) is determined by the Analytic Hierarchy Process (AHP) (Esra and Yasemin, 2004), the objective weight (*OW*) is determined by the Entropy Weight Method (EWM) (Jiang et al., 2018), and combined weight (*CW*) is determined by the Game Theory (GT) (Wang et al., 2020).
- Determine the first-level fuzzy evaluation model.

For the single-layer fuzzy evaluation of the factor set, the subfactors belonging to each factor set are used to calculate the fuzzy evaluation index of each factor set (Equation 3):

(3) $$b_{iz}=w_{ij}r_{jz}=\left[\begin{array}{ccc} w_{11} & w_{12} & \begin{array}{cc} \cdots & w_{1m} \end{array}\\ w_{21} & w_{22} & \begin{array}{cc} \cdots & w_{2m} \end{array}\\ \begin{array}{c} w_{31}\\ w_{41} \end{array} & \begin{array}{c} w_{32}\\ w_{41} \end{array} & \begin{array}{cc} \begin{array}{c} \cdots \\ \cdots \end{array} & \begin{array}{c} w_{3m}\\ w_{4m} \end{array} \end{array} \end{array}\right]\left[\begin{array}{ccc} r_{11} & r_{12} & \begin{array}{cc} \cdots & r_{1n} \end{array}\\ r_{21} & r_{22} & \begin{array}{cc} \cdots & r_{2n} \end{array}\\ \begin{array}{c} \vdots \\ r_{m1} \end{array} & \begin{array}{c} \vdots \\ r_{m2} \end{array} & \begin{array}{cc} \begin{array}{c} \ddots \\ \cdots \end{array} & \begin{array}{c} \vdots \\ r_{mn} \end{array} \end{array} \end{array}\right]=\left[\begin{array}{ccc} b_{11} & b_{12} & \begin{array}{cc} \cdots & b_{1n} \end{array}\\ b_{21} & b_{22} & \begin{array}{cc} \cdots & b_{2n} \end{array}\\ \begin{array}{c} b_{31}\\ b_{41} \end{array} & \begin{array}{c} b_{32}\\ b_{42} \end{array} & \begin{array}{cc} \begin{array}{c} \cdots \\ \cdots \end{array} & \begin{array}{c} b_{3n}\\ b_{4n} \end{array} \end{array} \end{array}\right]$$

Where $\sum_{\text{z}=1}^{\text{n}}{\text{b}}_{\text{iz}}=1,\;0\leq {\text{b}}_{\text{iz}}\leq 1$; In the formula, *b_iz_*is the fuzzy evaluation index of the *i*-th factor set; *w_ij_* is the OW of the *j*-th subfactor set under the *i*-th factor set; *r_jz_* is the normalized data matrix of the *i*-th factor set.

2. Establishment of the second-level fuzzy comprehensive evaluation model

- Determine the evaluation matrix of the evaluation factor set.
- Establish the weight set of the second-level evaluation factor set, taking the local weights obtained by the AHP as the standard.
- Determine the second-level comprehensive evaluation model:

For the secondary fuzzy evaluation of each treatment: the final fuzzy evaluation index of each factor set is used to calculate the comprehensive fuzzy evaluation index of all treatments (Equation 4).

(4) $$B_{z}=a_{i}b_{iz}=\left[\begin{array}{ccc} a_{1} & \begin{array}{cc} a_{2} & a_{3} \end{array} & a_{4} \end{array}\right]\left[\begin{array}{ccc} b_{11} & b_{12} & \begin{array}{cc} \cdots & b_{1n} \end{array}\\ b_{21} & b_{22} & \begin{array}{cc} \cdots & b_{2n} \end{array}\\ \begin{array}{c} b_{31}\\ b_{41} \end{array} & \begin{array}{c} b_{32}\\ b_{42} \end{array} & \begin{array}{cc} \begin{array}{c} \cdots \\ \cdots \end{array} & \begin{array}{c} b_{3n}\\ b_{4n} \end{array} \end{array} \end{array}\right]=\left[\begin{array}{ccc} B_{1} & B_{2} & \begin{array}{cc} \cdots & B_{n} \end{array} \end{array}\right]$$

where $\sum_{\text{z}=1}^{\text{n}}{\text{B}}_{\text{z}}=1,\;0\leq {\text{B}}_{\text{z}}\leq 1$. In the formula, *B_z_* is the multi-level fuzzy evaluation index of the *z*-th treatment; *a_i_* is the weight of the *i*-th factor set.

3. Analysis of the second-level comprehensive evaluation results

According to the principle of maximum membership degree, the comprehensive evaluation index corresponding to the largest evaluation indicator in *B_z_* is selected as the evaluation result.

#### 2.3.6 Particle swarm optimization algorithm

The PSO originated from the study of the foraging behavior of bird flocks during flight. During the process of searching for food, bird flocks transmit their positions to the flock through information exchange. The entire bird flock, through collaborative cooperation, finds the area around the food source and judges the position of the food (Feng et al., 2021). When the particle swarm optimization algorithm solves optimization problems, the ‘birds’ are also called ‘particles’. During the search process of particles in the feasible solution space, the values of ‘current individual optimal position’ and ‘global particle optimal position’ are updated according to the fitness value (Xu et al., 2013). In the two-dimensional space solution of this study, the initial population size is set to 30. Let ${\text{X}}_{\text{i}}={\left({\text{x}}_{\text{i}1},{\text{x}}_{\text{i}2}\right)}^{\text{T}}$ represent the position of the *i*-th particle, ${\text{P}}_{\text{best}}={\text{P}}_{\text{i}}={\left({\text{p}}_{\text{i}1},{\text{p}}_{\text{i}2}\right)}^{\text{T}}$ represent the individual optimal position searched by the particle, and ${\text{G}}_{\text{best}}={\text{P}}_{\text{g}}={\left({\text{p}}_{\text{g}1},{\text{p}}_{\text{g}2}\right)}^{\text{T}}$ represent the global optimal position searched by the entire population. Particles determine the global optimal solution of the function by continuously searching for the two optimal positions. The velocity update formula and position update formula of particles are shown in Equations 5, 6 respectively:

(5) $$V_{i}^{t+1}=\omega \cdot V_{i}^{t}+c_{1}\cdot r_{1}\cdot \left(P_{i}^{t}-X_{i}^{t}\right)+c_{2}\cdot r_{2}\cdot \left(P_{g}^{t}-X_{i}^{t}\right)$$

(6) $$X_{i}^{t+1}=X_{i}^{t}+V_{i}^{t+1}$$

where *ω* is the inertia weight, generally taken as 0.7; ${\text{V}}_{\text{i}}^{\text{t}}$ represents the flight velocity of the *i*-th particle, *i* = 1, 2, ⋯, 30; *t* is the current iteration number, and the iteration number is set to 50. Each iteration traverses the particles in the population; *c*_1_ and *c*_2_ are acceleration constants, also known as learning factors, generally taking values ranging from 1.4 to 2.0. In this study, 2 is taken; *r*_1_ and *r*_2_ represent random numbers in the range of [0, 1]. The velocity and position of each particle (solution) are randomly initialized in the search space, and the global optimal position is finally determined by calculating the fitness value of the function. To prevent particles from exceeding the value range of the problem solution during the search process, the following boundary restrictions are imposed on the particle velocity and position (Equations 7, 8):

(7) $$\text{Boundary bounce},x_{ij}^{t}=\{\begin{array}{ll} {\text{x}}_{\text{ij}}^{\text{t}}-{\text{X}}_{\max}\text{ if} & {\text{x}}_{\text{ij}}^{\text{t}}>1\\ x_{ij}^{t}+{\text{X}}_{\max\;}if & x_{ij}^{t}<0\\ x_{ij}^{t} & \text{otherwise} \end{array}$$

(8) $$\text{Boundary absorption},x_{ij}^{t}=\{\begin{array}{ll} 1 & if\;x_{ij}^{t}>1\\ 0 & if\;x_{ij}^{t}<0\\ x_{ij}^{t} & \text{otherwise} \end{array}$$

### 2.4 Data processing and analysis

**Figure 1:**
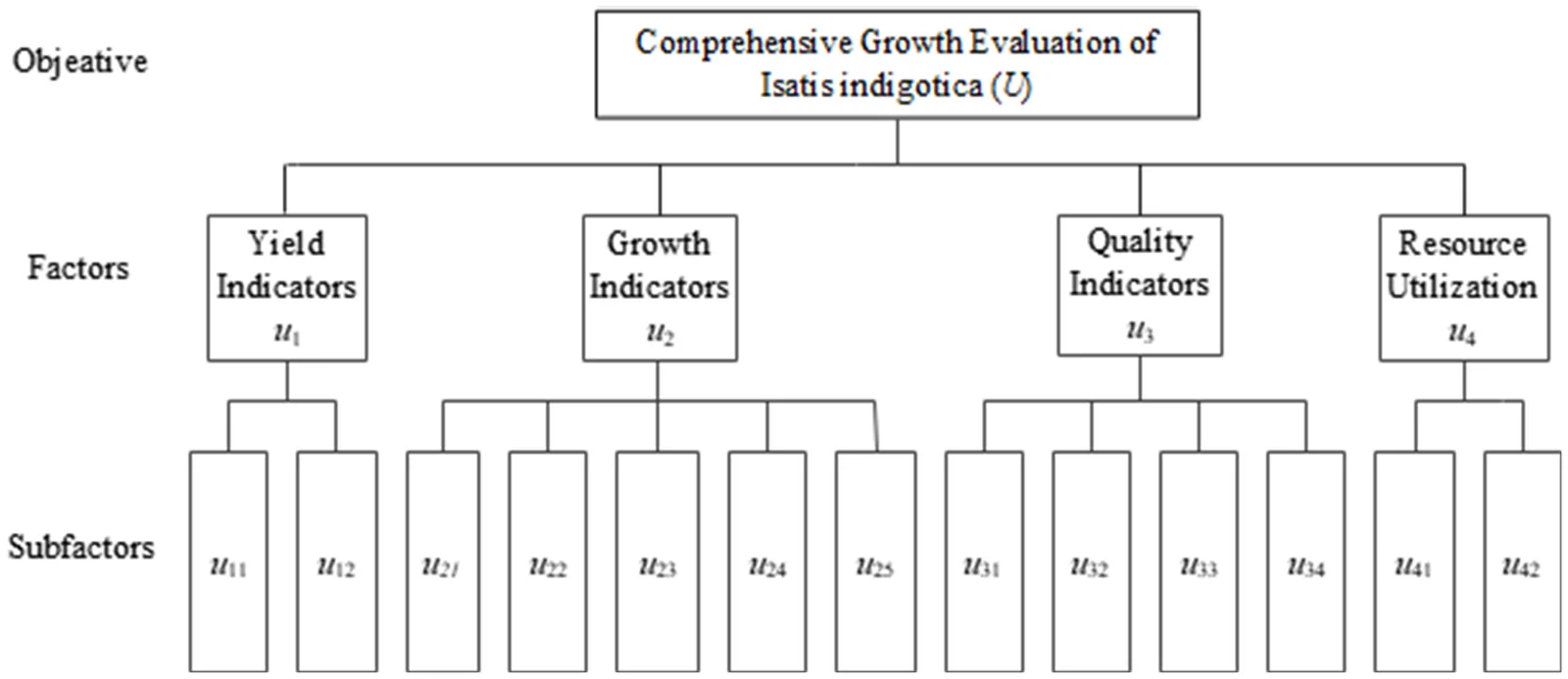
Comprehensive growth evaluation system of *Isatis indigotica*. *u*_11_: Root dry matter; *u*_12_: Economic yield; *u*_21_: Plant height; *u*_22_: Net photosynthetic rate; *u*_23_: Leaf area index; *u*_24_: Root length; *u*_25_: Root diameter; *u*_31_: Indigo; *u*_32_: Indirubin; *u*_33_: Epigoitrin; *u*_34_: Polysaccharide; *u*_41_: WUE; *u*_42_: NFPP.

The Microsoft Excel 2024 (Microsoft Corp., Raymond, Washington, USA) software was used for data organization and tabulation. The SPSS 25.0 (IBM, Inc., New York, USA) software was used for regression modeling and solution. Yaahp 10.01 (Developed by Shanxi Yuan-decision Software Technology Co., Ltd., China) was used to hierarchically classify the 4 types of attribute indicators of *Isatis indigotica* (**Figure 1**); Matlab R2025a was used to mathematically simulate the evaluation index; and the Origin2021 (Origin Lab, Corp., Hampton, Massachusetts, USA) software was used to draw relevant charts.

## 3 Results

### 3.1 Establishment of the comprehensive growth evaluation system for *Isatis indigotica*

To enable the comprehensive growth evaluation system of *Isatis indigotica* to rationally evaluate water-nitrogen supply strategies, considering economic, social, and ecological aspects, it is divided into four types of attribute indicators: growth, yield, quality, and resource utilization, resulting in the comprehensive growth evaluation system diagram (**Figure 1**). These four types of indicators are defined as the factor set *U* = {*u*_1_, *u*_2_, *u*_3_, *u*_4_} = {yield indicators, growth indicators, quality indicators, resource utilization indicators}. Meanwhile, the 13 secondary indicators belonging to the above-mentioned four major categories of indicators are defined as subfactors. Among them, those classified under the yield indicators (*u*_1_) are: root dry matter (*u*_11_) and economic yield (*u*_12_); those classified under the growth indicators (*u*_2_) are: plant height (*u*_21_), net photosynthetic rate (*u*_22_), leaf area index (*u*_23_), root length (*u*_24_), and root diameter (*u*_25_); those classified under the quality indicators (*u*_3_) are: indigo (*u*_31_), indirubin (*u*_32_), epigoitrin (*u*_33_), and polysaccharide (*u*_34_); those classified under the resource utilization indicators (*u*_4_) are: WUE (*u*_41_) and NFPP (*u*_42_). In addition, the sets of evaluation values corresponding to each factor and its subfactors are constructed, generally represented by *V* and *v_ij_*. Since there are 10 treatments in this study, there are 10 corresponding evaluation values.

### 3.2 Calculation of weights

The AHP establishes a judgment matrix based on the results of a questionnaire. The overall decision-making is divided into the target layer, factor layer, and sub-factor layer. By comparing the importance of the indicators of each subfactor set (factor set indicators), a comparison matrix is established, and the subfactors are scored using the questionnaire. The values of the indicators in the comparison matrix are generally determined by the 1–9 scale method. The consistency of the comparison matrix is tested to determine its acceptability. When the consistency ratio index *CR<* 0.1, the consistency test is considered passed, and the indicator judgment matrix is acceptable. In this study, the comparison matrices of the factor layer and subfactor layer indicators are shown in **Table 2** as follows. Yaahp10.01 is used to process the scores of all factors and subfactors, and then the SW values corresponding to the factor set and subfactor set are obtained, respectively. The specific results are shown in **Table 2**. The weight vector is solved using the eigenvalue method, and all results pass the consistency test, that is, *CR<* 0.1. Among the weight results of the factor set indicators of *Isatis indigotica*, the yield indicator has the largest weight (0.3584), followed by the quality indicators and the growth indicators, and the resource utilization indicators have the smallest weight, which is 0.1121. Among the weight results of the subfactor set indicators, the maximum value is for the economic yield (*EY*), which is 0.2389, followed by the root dry matter (*RDM*) weight, which is 0.135, and the plant height (*PH*) has the smallest weight, only 0.0237.

**Table 2:** Indicator weights obtained based on the AHP method. *a*_1_ is the weight of yield indicators; *a*_2_ is the weight of growth indicators; *a*_3_ is the weight of quality indicators; *a*_4_ is the weight of resource utilization indicators.

Indicators	Comparison matrix	Local weight *W_ij_*	Final weight *a_ij_*	Consistency test parameter
Target layer *U* Factor layer *u_i_*	${\text{u}}_{\text{i}}=\left[\begin{array}{cccc} 1 & 2 & 1 & 3\\ 1/2 & 1 & 1 & 3\\ 1 & 1 & 1 & 2\\ 1/3 & 1/3 & 1/2 & 1 \end{array}\right]$	0.3584	0.3584(*a*_1_)	*CR* = 0.0305 *λ_max_* = 4
		0.2566	0.2566(*a*_2_)	
		0.2729	0.2729(*a*_3_)	
		0.1121	0.1121(*a*_4_)	
Factor layer *u*_1_ Subfactor layer *u*_1_*_j_*	${\text{u}}_{1\text{j}}=\left[\begin{array}{cc} 1 & 1/2\\ 2 & 1 \end{array}\right]$	0.3333	0.1195	*CR* = 0.0000 *λ_max_* = 2
		0.6667	0.2389	
Factor layer *u*_2_ Subfactor layer *u*_2_*_j_*	${\text{u}}_{2\text{j}}=\left[\begin{array}{ccccc} 1 & 1/2 & 1/2 & 1/3 & 1/4\\ 2 & 1 & 1 & 2 & 2\\ 2 & 1 & 1 & 3 & 3\\ 3 & 1/2 & 1/3 & 1 & 1/2\\ 4 & 1/2 & 1/3 & 2 & 1 \end{array}\right]$	0.0922	0.0237	*CR* = 0.0953 *λ_max_* = 5
		0.2609	0.0669	
		0.3145	0.0807	
		0.1384	0.0355	
		0.1939	0.0498	
Factor layer *u*_3_ Subfactor layer *u*_3_*_j_*	${\text{u}}_{3\text{j}}=\left[\begin{array}{cccc} 1 & 1/2 & 1/2 & 1/3\\ 2 & 1 & 2 & 1\\ 2 & 1/2 & 1 & 1\\ 3 & 1 & 1 & 1 \end{array}\right]$	0.1257	0.0343	*CR* = 0.0304 *λ_max_* = 4
		0.3319	0.0906	
		0.2347	0.0641	
		0.3076	0.0840	
Factor layer *u*_4_ Subfactor layer *u*_4_*_j_*	${\text{u}}_{4\text{j}}=\left[\begin{array}{cc} 1 & 3\\ 1/3 & 1 \end{array}\right]$	0.7500	0.0841	*CR* = 0.0000 *λ_max_* = 2
		0.2500	0.0280	

*λ_max_*, Maximum characteristic root of the judgment matrix; *CR*, Consistency ratio for consistency check.

The EWM calculates weights using the standardized measured values. Considering the relationships among multiple samples, it can make the evaluation results of multi-sample data more objective and reasonable. The *OW* values of the subfactor set indicators calculated based on the measured statistical values are shown in **Table 2**. To improve the scientificity of the weight assignment of the participating factors and the reliability of the evaluation conclusion, the GT combination weight-assignment method is used for the subfactor set indicators participating in the first-level fuzzy evaluation. That is, the local *SW* obtained by the AHP method and the *OW* obtained by the EWM method are balanced to obtain a unified subjective combination weight. The specific results are shown in **Table 3** as follows.

**Table 3:** Subjective, objective weights, and their combined weights of subfactor-set indicators.

Year	Weight	*u*_1_		*u*_2_					*u*_3_				*u*_4_	
		*u*_11_	*u*_12_	*u*_21_	*u*_22_	*u*_23_	*u*_24_	*u*_25_	*u*_31_	*u*_32_	*u*_33_	*u*_34_	*u*_41_	*u*_42_
2018	*OW*	0.5065	0.4935	0.2210	0.2108	0.2061	0.1723	0.1899	0.2279	0.3241	0.2289	0.2191	0.6591	0.3409
	*SW*	0.3333	0.6667	0.0922	0.2609	0.3145	0.1384	0.1939	0.1257	0.3319	0.2347	0.3076	0.7500	0.2500
		*w*_11_	*w*_12_	*w*_21_	*w*_22_	*w*_23_	*w*_24_	*w*_25_	*w*_31_	*w*_32_	*w*_33_	*w*_34_	*w*_41_	*w*_42_
	*CW*	0.5001	0.4999	0.2184	0.2118	0.2083	0.1716	0.1900	0.2276	0.3241	0.2289	0.2193	0.5000	0.5000
2019	*OW*	0.5013	0.4987	0.2175	0.2113	0.2536	0.1509	0.1667	0.2335	0.2981	0.2465	0.2219	0.6447	0.3553
	*SW*	0.3333	0.6667	0.0922	0.2609	0.3145	0.1384	0.1939	0.1257	0.3319	0.2347	0.3076	0.7500	0.2500
		*w*_11_	*w*_12_	*w*_21_	*w*_22_	*w*_23_	*w*_24_	*w*_25_	*w*_31_	*w*_32_	*w*_33_	*w*_34_	*w*_41_	*w*_42_
	*CW*	0.5000	0.5000	0.2248	0.2084	0.2501	0.1516	0.1651	0.2389	0.2964	0.2471	0.2176	0.5000	0.5000

*OW*, Objective weight; *SW*, Subjective weight; *CW*, Combined weight. *u*_1_, yield indicators; *u*_2_, growth indicators; *u*_3_, quality indicators; *u*_4_, resource utilization indicators. *u*_11_, Root dry matter; *u*_12_, Economic yield; *u*_21_, Plant height; *u*_22_, Net photosynthetic rate; *u*_23_, Leaf area index; *u*_24_, Root length; *u*_25_, Root diameter; *u*_31_, Indigo; *u*_32_, Indirubin; *u*_33_, Epigoitrin; *u*_34_, Polysaccharide; *u*_41_, WUE; *u*_42_, NFPP.

### 3.3 Multi-level fuzzy comprehensive evaluation model

#### 3.3.1 First-level fuzzy comprehensive evaluation

1. Fuzzy comprehensive evaluation of yield and resource utilization indicators

**Figure 2:**
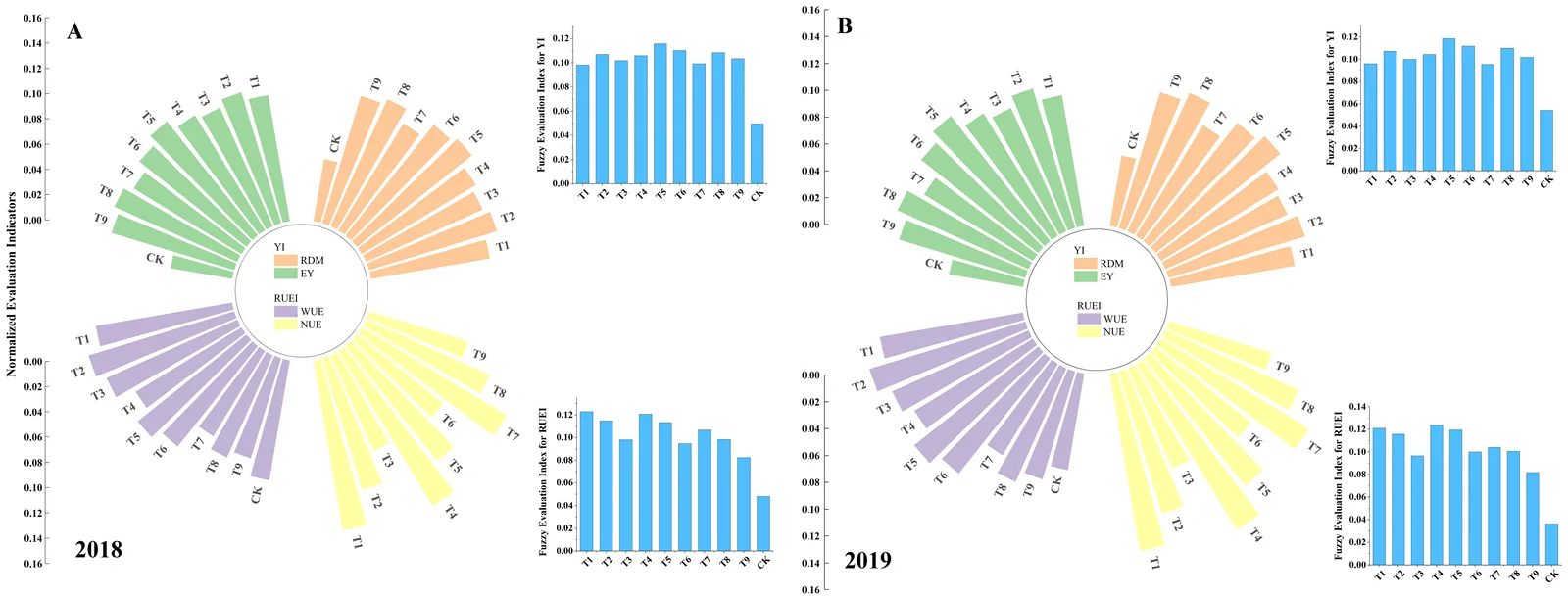
Fuzzy evaluation indexes of yield and resource utilization indicators of Isatis indigotica in 2018 **(A)** and 2019 **(B)**. RDM, Root dry matter; EY, Economic yield; WUE, Water use efficiency; NFPP, Nitrogen fertilizer partial productivity; YI, Yield indicators; RUI, Resource utilization indicators.

Based on the first-level fuzzy comprehensive evaluation, the yield indicators and resource utilization indicators of *Isatis indigotica* were evaluated. The yield indicators mainly include *RDM* and *EY*, while the resource utilization indicators mainly include *WUE* and *NFPP*. After standardizing the data, the evaluation values of the yield indicators and resource utilization were obtained, and after assigning the combined weights, the first-level fuzzy evaluation indexes of each treatment were obtained (**Figure 2**). As can be seen from **Figure 1**, regarding the influence of different irrigation levels and nitrogen application levels on the yield and resource utilization indicators of *Isatis indigotica*, the standardized evaluation values of *RDM* and *EY* of treatment T5 were the largest. In 2018, they were 0.1162 and 0.1154, respectively, and in 2019, they were 0.1185 and 0.1189, respectively, while those of the control CK were the smallest, being 0.0497 and 0.0495 in 2018, and 0.0530 and 0.0558 in 2019, respectively. The standardized evaluation value of *WUE* of treatment T2 was the largest, being 0.1204 in 2018 and 0.1212 in 2019, while that of treatment T7 was the smallest in 2018, being 0.0790, and that of the control CK was the smallest in 2019, being 0.0727. The standardized evaluation value of *NFPP* of treatment T4 was the largest, being 0.1436 and 0.1446 in 2018 and 2019, respectively, while that of treatment T9 was the smallest, being 0.0819 and 0.0799, respectively. At the same irrigation level, the standardized evaluation values of *RDM*, *EY*, and *WUE* first increased and then decreased with the increase of nitrogen application rate, that is, N2 > N3 > N1, while the standardized evaluation value of *NFPP* decreased continuously, that is, N1 > N2 > N3. At the same nitrogen application level, the standardized evaluation values of *RDM*, *EY*, and *NFPP* also first increased and then decreased with the increase of irrigation amount, that is, W2 > W1 and W2 > W3, but the standardized evaluation value of *WUE* decreased continuously, that is, W1 > W2 > W3. From the perspective of the first-level fuzzy evaluation index, the yield index (*YI*) was still the largest for treatment T5, being 0.1158 and 0.1187 in the two years, respectively, while that of the control CK was the smallest, being 0.0496 and 0.0544, respectively. For the resource utilization index (*RUI*), in 2018, treatment T1 had the largest value, being 0.1229, followed by treatment T4, being 0.1208. In 2019, treatment T4 had the largest value, being 0.1239, and treatment T1 was the second, being 0.1210, while the smallest was still the control CK, being 0.0482 and 0.0364, respectively. In addition, at the same irrigation level, the *YI* of the low-nitrogen and high-nitrogen treatments was lower than that of the medium-nitrogen treatment, and the *RUI* decreased with the increase of nitrogen application rate. At the same nitrogen application level, the *YI* of the medium-water treatment was higher than that of the low-water and high-water treatments, and the *RUI* of the low-water and medium-water treatments was higher than that of the high-water treatment.

2. Fuzzy comprehensive evaluation of growth indicators

**Figure 3:**
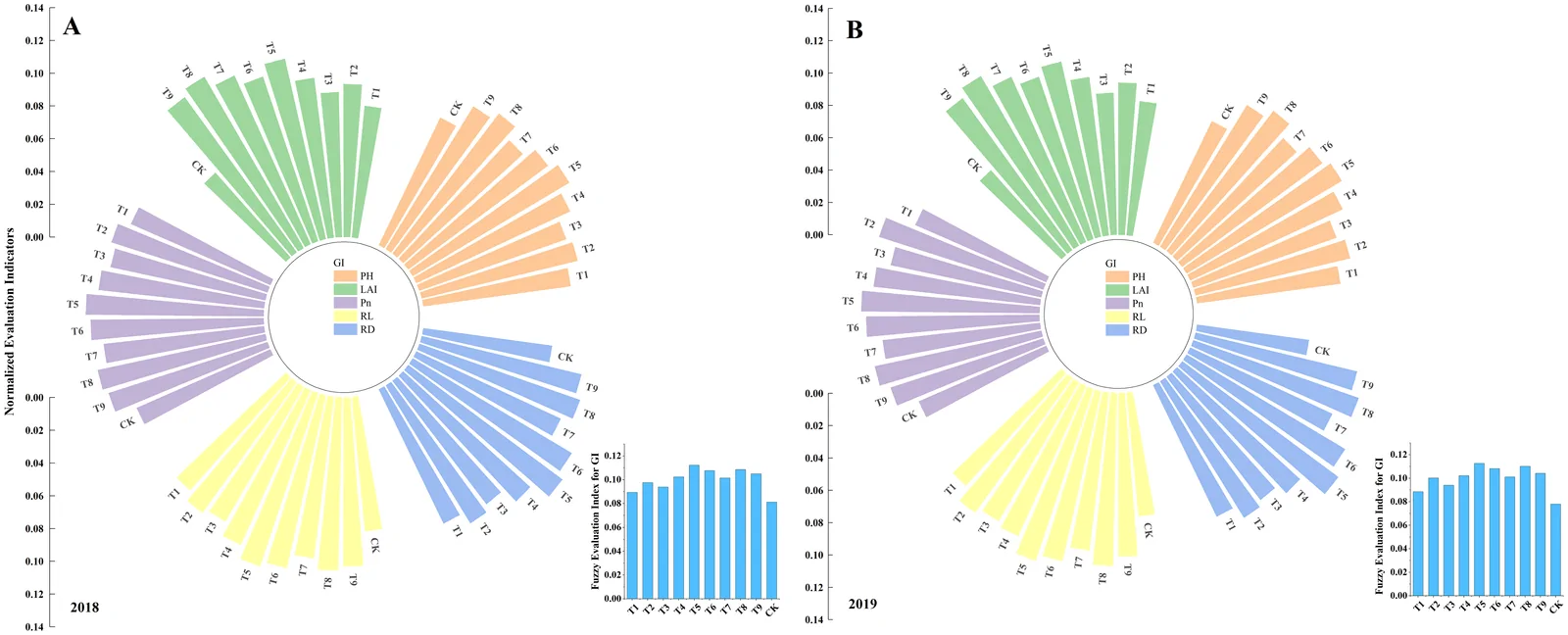
Fuzzy evaluation indexes of growth indicators of Isatis indigotica in 2018 **(A)** and 2019 **(B)**. PH, Plant height; LAI, Leaf area index; Pn, Net photosynthetic rate; RL, Root length; RD, Root diameter; GI, Growth indicators.

Based on the first-level fuzzy comprehensive evaluation, the growth indicators of *Isatis indigotica* were evaluated. The growth indicators mainly include plant height (*PH*), leaf area index (*LAI*), net photosynthetic rate (*Pn*), root length (*RL*), and root diameter (*RD*). After standardizing the data, the evaluation values of the growth indicators were obtained, and after assigning the combined weights, the first-level fuzzy evaluation indexes of each treatment were obtained (**Figure 3**). As can be seen from **Figure 2**, regarding the influence of different irrigation levels and nitrogen application levels on the growth indicators of *Isatis indigotica*, the standardized evaluation values of *PH*, *Pn*, *RL*, and *RD* of treatment T5 were the largest. In 2018, they were 0.1114, 0.1086, 0.1115, and 0.1173, respectively, and in 2019, they were 0.1118, 0.1103, 0.1121, and 0.1202, respectively, while those of the control CK were the smallest, being 0.0864, 0.0891, 0.0824, and 0.0794 in 2018, and 0.0835, 0.0860, 0.0768, and 0.0699 in 2019, respectively. The standardized evaluation value of *LAI* of treatment T8 was the largest, being 0.1207 and 0.1211 in the two years, respectively, and the smallest was also CK, being 0.0691 and 0.0700, respectively. At the same irrigation level, the standardized evaluation values of *PH*, *LAI*, *Pn*, *RL*, and *RD* first increased and then decreased with the increase of nitrogen application rate, that is, N2 > N3 > N1. At the same nitrogen application level, the standardized evaluation values of *PH*, *Pn*, *RL*, and *RD* also first increased and then decreased with the increase of irrigation level, that is, W2 > W3 > W1, but the standardized evaluation value of *LAI* increased continuously, that is, W3 > W2 > W1. From the perspective of the first-level fuzzy evaluation index of the growth indicators, treatment T5 was the largest, being 0.1123 and 0.1127 in the two years, respectively, followed by treatment T8, and the control CK was the smallest, being 0.0813 and 0.0780, respectively. In addition, at the same irrigation level, *GI* increased with the increase of nitrogen application rate in the order of N2 > N3 > N1. At the same nitrogen application level, *GI* also showed the same pattern, that is, W2 > W3 > W1.

3. Fuzzy comprehensive evaluation of quality indicators

**Figure 4:**
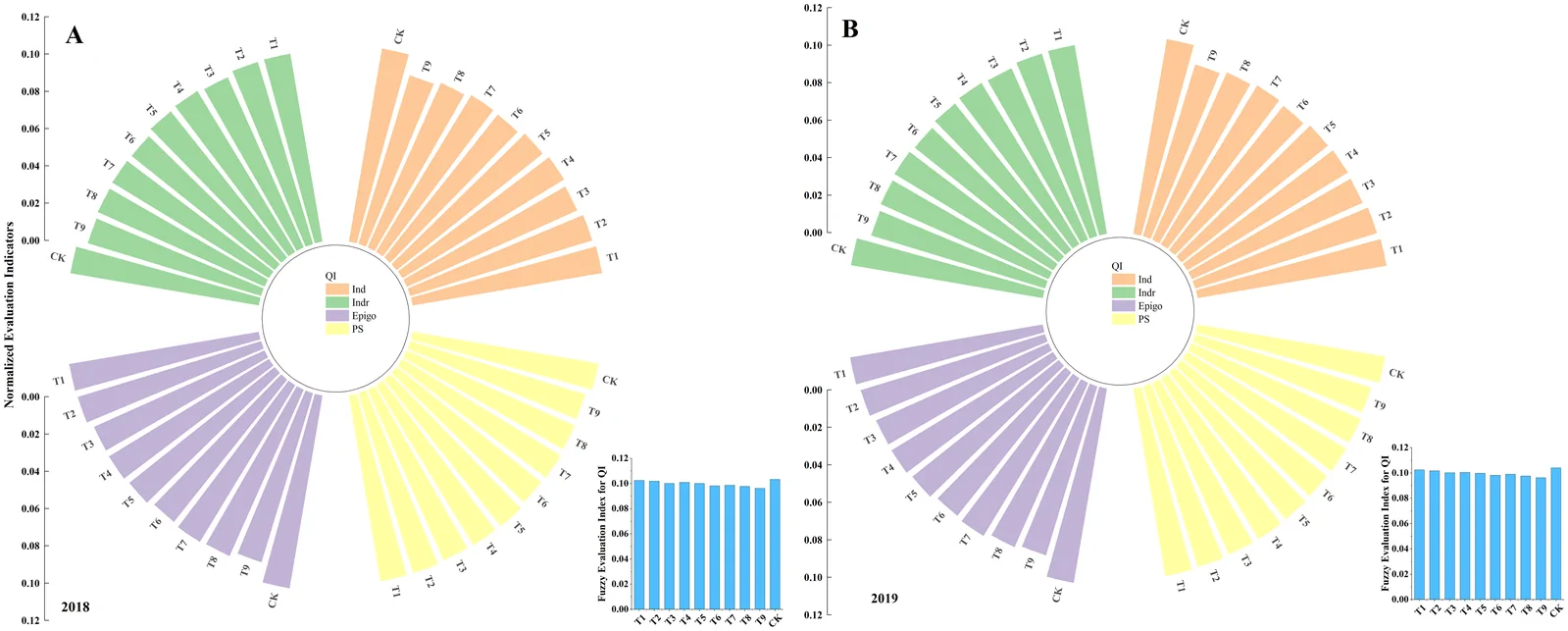
Fuzzy evaluation indexes of quality indicators of Isatis indigotica in 2018 **(A)** and 2019 **(B)**. Ind, Indigo; Indr, Indirubin; Epigo, Epigoitrin; PS, Polysaccharides; QI, Quality indicators.

Based on the first-level fuzzy evaluation, the quality indicators of *Isatis indigotica* were evaluated. The quality indicators mainly include indigo (*Ind*), indirubin (*Indr*), (R, S)-epigoitrin (*Epigo*), and polysaccharide (*PS*). After standardizing the data, the evaluation values of the quality indicators were obtained, and after assigning the combined weights, the first-level fuzzy evaluation indexes of each treatment were obtained (**Figure 4**). As can be seen from **Figure 3**, among the water-nitrogen treatments, the standardized evaluation values of *Ind*, *Indr*, *Epigo*, and *PS* of treatment T1 were the largest. In 2018, they were 0.1033, 0.1023, 0.1033, and 0.1025, respectively, and in 2019, they were 0.1024, 0.1021, 0.1042, and 0.1013, respectively, while those of treatment T9 were the smallest, being 0.0945, 0.0972, 0.0983, and 0.0963 in 2018, and 0.0957, 0.0970, 0.0942, and 0.0981 in 2019, respectively. At the same irrigation level, the standardized evaluation values of each quality indicator decreased with the increase of nitrogen application rate, that is, N1 > N2 > N3. At the same nitrogen application level, the standardized evaluation values of each quality indicator also decreased with the increase of irrigation level, that is, W1 > W2 > W3. From the perspective of the first-level fuzzy evaluation index of the quality indicators, among the water-nitrogen treatments, the *QI* of the low-water and low-nitrogen treatment was the largest, being 0.1025 in both years, followed by the low-water and medium-nitrogen treatment, and the high-water and high-nitrogen treatment was the smallest, being 0.0963 and 0.0962 in the two years respectively. In addition, at the same irrigation level, *QI* decreased with the increase of nitrogen application rate in the order of N1 > N2 > N3. At the same nitrogen application level, *QI* decreased with the increase of irrigation level in the order of W1 > W2 > W3.

In summary, from the results of the first-level fuzzy comprehensive evaluation, different water-nitrogen treatments cannot take into account all indicators. Among them, the medium-water and medium-nitrogen treatment (T5) had relatively good yield and growth, the low-water or medium-water and low-nitrogen treatment (T1 or T4) had relatively good resource utilization, and the low-water treatment (W1) had relatively good quality. It can be seen that the first-level evaluation cannot take into account all types of attribute indicators, and further consideration is needed on this basis.

#### 3.3.2 Second-level fuzzy comprehensive evaluation

**Figure 5:**
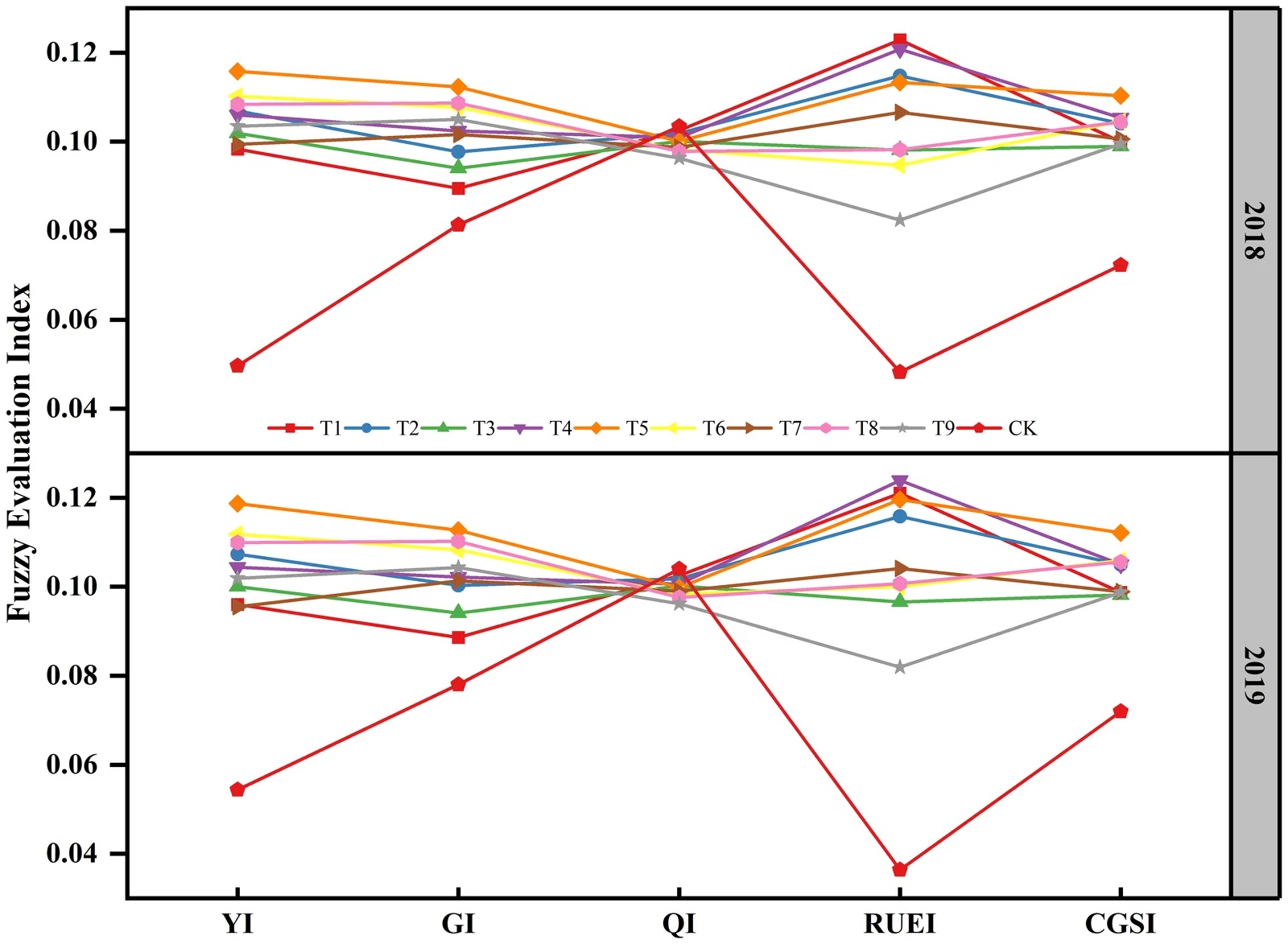
Second-level fuzzy comprehensive evaluation index. YI, Yield indicators; GI, Growth indicators; QI, Quality indicators; RUI, Resource utilization indicators; CGSI, Comprehensive growth of Isatis indigotica.

According to the comprehensive growth evaluation system of *Isatis indigotica*, the participating evaluation indicators of the second-level fuzzy comprehensive evaluation include yield indicators, growth indicators, quality indicators, and resource utilization indicators. The results of the first-level fuzzy comprehensive evaluation were used as the single-factor evaluation matrix of the second-level evaluation. The final weights *a_i_* = (0.3584, 0.2566, 0.2729, 0.1121) are calculated by the AHP method, where the values correspond to the yield, growth, quality, and resource utilization indicators in sequence. After weighting, the second-level fuzzy evaluation index *B_z_* was obtained, and the treatments were sorted accordingly. The final results of the comprehensive growth evaluation of *Isatis indigotica* are shown in **Figure 4**. As can be seen from **Figure 5**, the multi-level fuzzy evaluation indexes of different treatments were ranked as follows: in 2018, T5 > T4 > T6 > T8 > T2 > T7 > T1 > T9 > T3 > CK; in 2019, T5 > T6 > T8 > T4 > T2 > T7 > T9 > T1 > T3 > CK.

From the above evaluation results, it can be seen that among the water-nitrogen treatments, the medium-water and medium-nitrogen treatments had the highest evaluation index. This is mainly because the growth of *Isatis indigotica* was relatively good, the yield was high, and the resource utilization efficiency was not bad. Under the condition of not overly favoring quality, the comprehensive growth benefit of this treatment of *Isatis indigotica* was relatively the best. The evaluation index of the low-water and high-nitrogen treatment was the lowest, mainly because except for relatively acceptable quality and yield, the other indicators of this treatment were relatively low.

### 3.4 Water-nitrogen decision-making for *Isatis indigotica*

**Figure 6:**
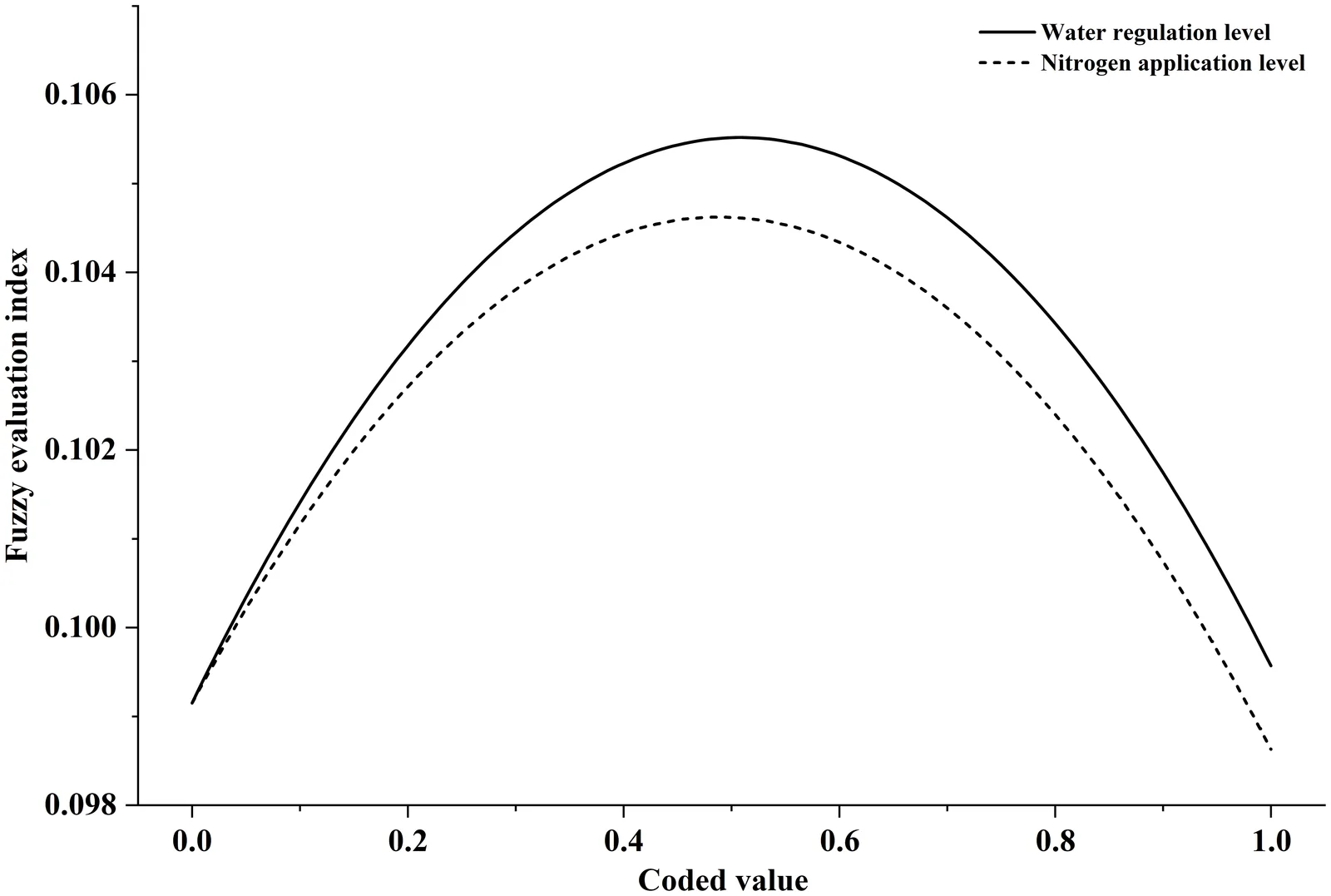
Regulatory effects of water-nitrogen interaction on the fuzzy evaluation index of *Isatis indigotica*.

By performing a step-by-step quadratic polynomial regression fitting on the second-level fuzzy evaluation indexes of *Isatis indigotica* under different water-nitrogen levels over two years, the regression equation (Equation 9) of the evaluation index with respect to the water-nitrogen levels is obtained (**Figure 6**):

(9) $$Y^{*}=-0.02463x_{1}^{2\;}+0.02505x_{1}-0.02292x_{2}^{2}\;+0.02240x_{2}+0.00017x_{1}x_{2}+0.09915$$

where *Y*^∗^ is the second-level fuzzy evaluation index of the growth, yield, quality, and resource utilization indicators of *Isatis indigotica* under water-nitrogen levels; *x*_1_ and *x*_2_ are the coded values of the irrigation level and nitrogen application level, ranging from 0 to 1.

A significance test was carried out on the regression equation. The correlation coefficient *R* = 0.988 and the determination coefficient *R*^2^ = 0.976, indicating a high degree of model fitting. The *F*-value is 97.682 and *P<* 0.01, suggesting that the regression relationship is at an extremely significant level, which proves that the established regression model is reliable, that is, water-nitrogen regulation has a significant impact on the fuzzy comprehensive evaluation results.

#### 3.4.1 Regulatory effects of water-nitrogen on the comprehensive growth of *Isatis indigotica*

To explore the impact of single water or nitrogen factors on the second-level fuzzy evaluation index of *Isatis indigotica*, the above-mentioned model was reduced in dimension, and the single-factor effect function formulas of irrigation level (Equation 10) and nitrogen application level (Equation 11) on the second-level fuzzy evaluation index of *Isatis indigotica* were obtained:

(10) $$Y_{1}^{*}=-0.02463x_{1}^{2}+0.02505x_{1}+0.09915$$

(11) $$Y_{2}^{*}=-0.02292x_{2}^{2}+0.02240x_{2}+0.09915$$

Where $Y_{1}^{*}$ and $Y_{2}^{*}$ refer to the single-factor effect functions of the irrigation level and nitrogen application level on the comprehensive evaluation index of *Isatis indigotica*, respectively.

**Figure 7:**
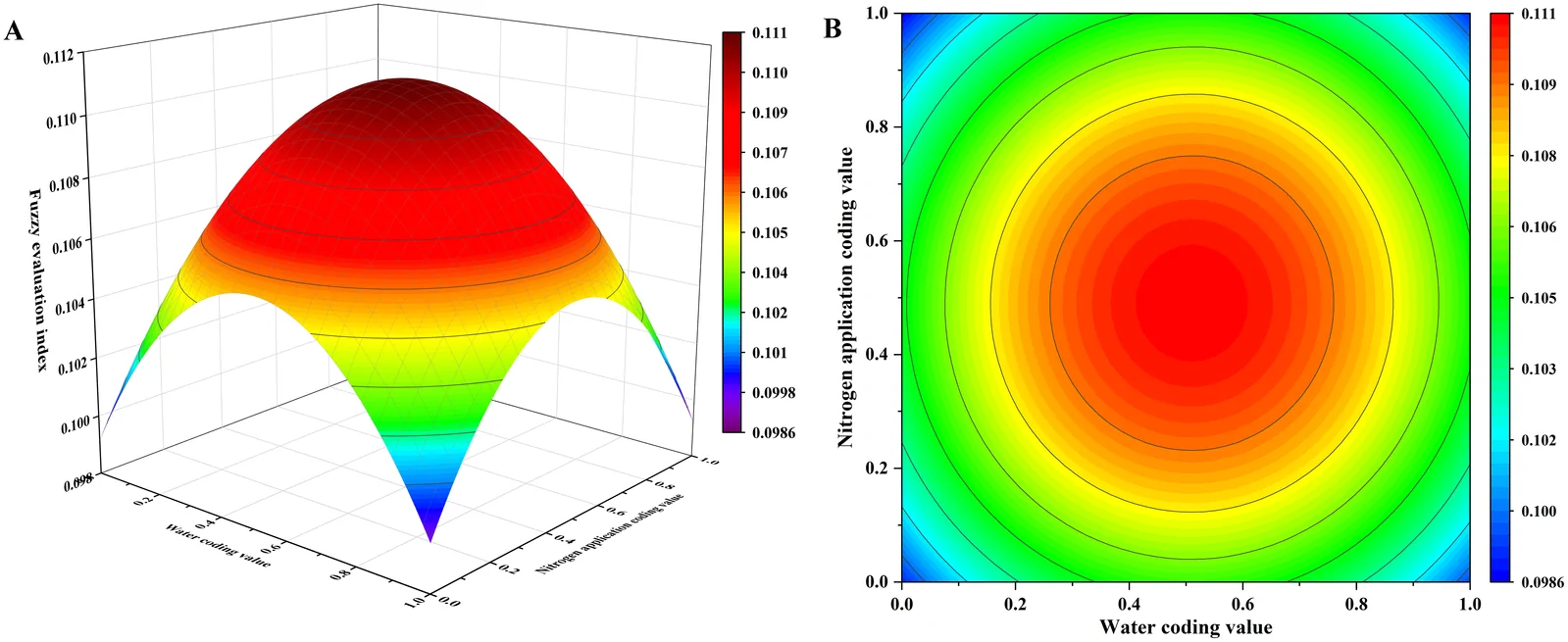
Regulatory effects of single water-nitrogen factors on the fuzzy evaluation index of *Isatis indigotica*. **(A)** is the algorithm flowchart; **(B)** is the graph of the fitness value changing with the number of iterations.

The impacts of irrigation level and nitrogen application level on the comprehensive growth of *Isatis indigotica* are shown in **Figure 7**. The second-level fuzzy evaluation index first increases and then decreases with the increase of water and nitrogen input. It can be seen that the two-factor of water and nitrogen follows the law of diminishing returns. When the coded value of the irrigation level *x*_1_ is 0.5085 and the coded value of the nitrogen application level *x*_2_ is 0.4887, the comprehensive evaluation value of *Isatis indigotica* is the largest.

#### 3.4.2 Optimal water-nitrogen levels of *Isatis indigotica* based on PSO

**Figure 8:**
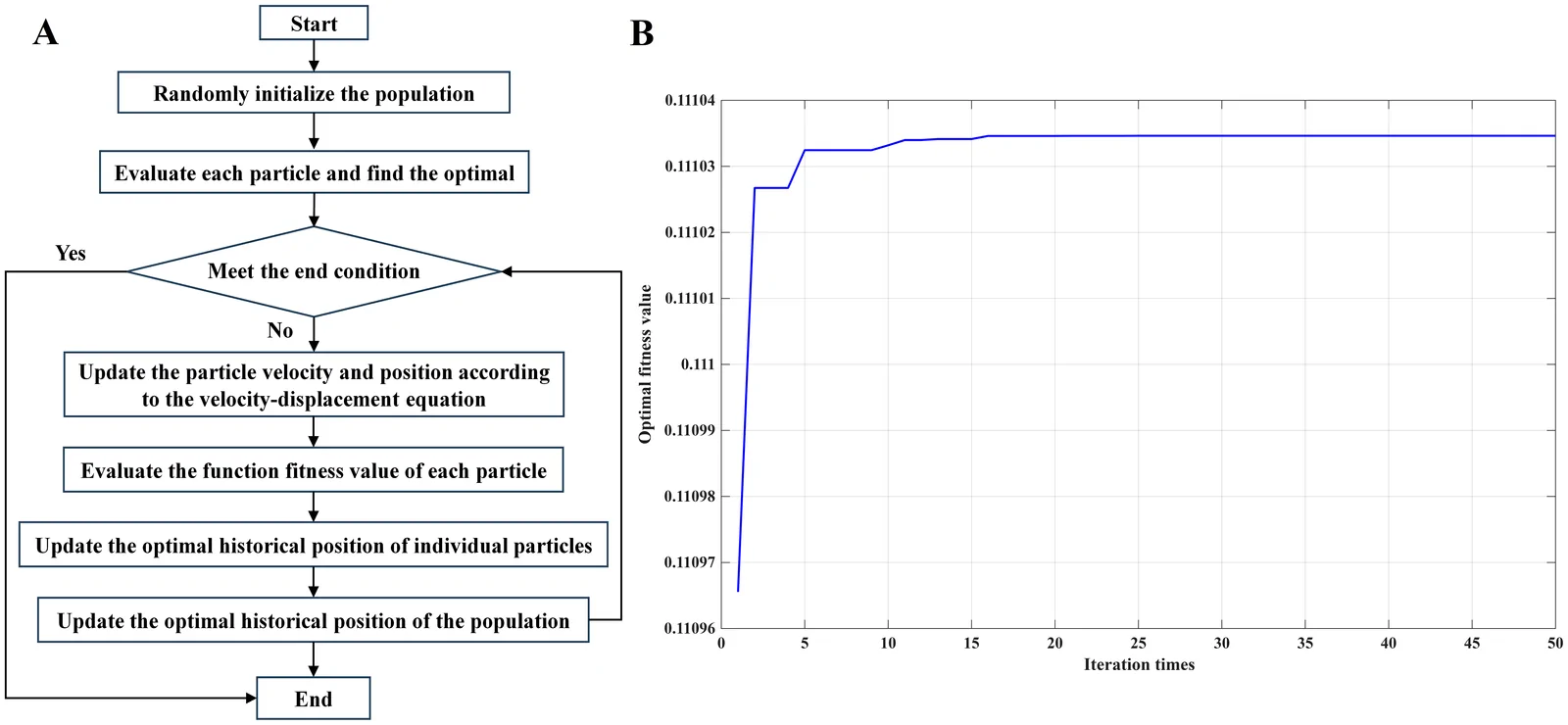
Flowchart and fitness curve of the particle swarm algorithm. Panel **(A)** shows the impact of water-nitrogen regulation on the evaluation index of *Isatis indigotica*; Panel **(B)** is the projection diagram.

Different water-nitrogen combinations of *Isatis indigotica* have a significant impact on its fuzzy comprehensive evaluation index. With the help of MATLAB software, the regression model was simulated and optimized based on the PSA with constraint boundaries, achieving a good convergence effect (**Figure 8**). When *x*_1_ = 0.5102 and *x*_2_ = 0.4906, the fitness function reaches the maximum value (absolute value). At this time, *Y*^∗^=0.1110, corresponding to the upper and lower limits of soil irrigation of 75.10%–85.10% and a nitrogen application rate of 224.53 kg·ha^-1^.

## 4 Discussion

### 4.1 Construction of the evaluation system and determination of indicator weights

The evaluation system is a complete organic system composed of evaluation dimensions, evaluation indicators, weight distribution rules, and evaluation models, etc. It is centered around specific evaluation objectives, relying on relevant theoretical foundations, and following a hierarchical, systematic, and standardized logic (Li et al., 2026). Based on the actual situation of agricultural production, following the principles of scientificity, systematicness, representativeness, etc., and combining the research results of crops such as grains (Zhao et al., 2021; Guo et al., 2025), vegetables (Zheng et al., 2022; Wang et al., 2026), and fruits and forest trees (Sun et al., 2024; Liu et al., 2025), this study screened 13 key evaluation indicators from four dimensions of the growth, yield, quality, and resource utilization of *Isatis indigotica*, and constructed a multi-level evaluation system including the target layer, factor set (criterion layer), and subfactor set (indicator layer). This system can objectively and comprehensively evaluate the comprehensive growth benefits of *Isatis indigotica* regulated by water-nitrogen. At the same time, indicator weights are quantitative coefficients that measure the relative importance and contribution of every single indicator in the evaluation system to the evaluation result, directly affecting the final decision-making (Kasim, 2014). Currently, there are many methods to determine weights, and most studies evaluate a single indicator through subjective or objective methods of determining weights (Tukino et al., 2024), while there are relatively few comprehensive evaluations of multiple indicators. In this study, based on considering the attributes of indicators and the characteristics of the model, subjective, objective, and combined methods of determining weights were adopted. That is, the AHP was used to determine the SW, including the final weights of the factor-set indicators and the local weights of each subfactor-set indicator. At the same time, based on the measured data, the EWM was used to obtain the OW of each subfactor-set indicator. Although Wang et al. (2021) used the AHP to determine the weights of the evaluation indicators for potato under water-fertilizer coupling, and Song et al. (2025) used the EWM to determine the weights of the evaluation indicators for tomato under water-fertilizer regulation, both achieved good evaluation results. However, in this study, to reduce subjectivity in the first-level fuzzy evaluation process, the GT combination method of determining weights was used for each subfactor-set indicator. This not only overcomes the shortcomings of a single weight but also ensures the reliability of the final evaluation result to a certain extent (Chen, 2003). In addition, the second-level fuzzy comprehensive evaluation is based on the results of the first-level fuzzy comprehensive evaluation. The weights of the factor-set indicators are obtained by the AHP. Although this ensures the actual needs of the model, the drawback is that the obtained weights can vary due to different subjective values.

### 4.2 Multi-level fuzzy evaluation of the comprehensive growth of *Isatis indigotica*

Fuzzy evaluation is based on the theory of fuzzy mathematics. Using the principle of fuzzy relation composition, it quantifies some factors with unclear boundaries and difficult quantification for evaluation (Xu et al., 2023). Based on the characteristics of multiple factors and fuzziness in the evaluation process, this study constructed a multi-level fuzzy evaluation model of the target, factor-set, and subfactor-set, and achieved a quantitative calculation of the comprehensive growth effect of *Isatis indigotica* through a layer-by-layer weighted aggregation method. He et al. (2021) also established a similar multi-level evaluation model and obtained good evaluation results. The first-level fuzzy comprehensive evaluation of the growth, yield, and resource utilization indicators of *Isatis indigotica* showed that the evaluation indices (*GI* and *YI*) of the growth and yield indicators of treatment T5 were the largest, while those of the control CK were the smallest. This is similar to the research conclusion of Zhang et al. (2017) on the water-fertilizer coupling experiment of *Isatis indigotica*, but it deviates greatly from the research conclusion of Zhang et al. (2016) on *Isatis indigotica*. This may be related to the variety selected for the experiment, the setting mode, and the climatic environment. For the resource utilization indicators, the evaluation indices (*RUI*) of treatment T1 and treatment T4 were relatively high, while the smallest was still the control CK. In addition, at the same irrigation level, the *GI* and *YI* of the medium-nitrogen treatment were relatively high, but the *RUI* decreased with the increase of the nitrogen application rate. At the same nitrogen application level, the *GI* and *YI* of the medium-water treatment were relatively high, while the *RUI* of the low-water and medium-water treatments were relatively high. The first-level fuzzy evaluation of the quality indicators of *Isatis indigotica* found that at the same irrigation level, *QI* decreased with the increase of nitrogen application rate. At the same nitrogen application level, *QI* also decreased with the increase of irrigation amount. Among them, the *QI* of treatment T1 was the largest, and that of treatment T9 was the smallest. Zhou et al. (2022) also obtained similar conclusions from the water deficit experiment of *Isatis indigotica*, that is, the fleshy root growth stage directly determines the formation of the quality of Banlangen medicinal materials. Mild water stress, without causing physiological damage to the plants, although the basic metabolic level slightly decreases, will trigger the plant’s stress signal response, regulate the synthesis of secondary metabolism related enzymes, promote the large scale conversion of primary metabolic products into medicinal secondary metabolic substances and continuous enrichment, thereby increasing the accumulation of effective components inside the medicinal materials (Shahrajabian et al., 2023). In summary, different water-nitrogen treatments cannot take all indicators into account. It is difficult to comprehensively represent the growth effect of *Isatis indigotica* relying on a single indicator. At the same time, there is an information overlapping phenomenon among various types of indicators, and each has its own unique evaluation value and cannot replace each other. This is similar to the evaluation result of He et al. (He et al., 2019) on the water regulation indicators of corn. Therefore, it is necessary to integrate multiple types of evaluation indicators for systematic analysis to ensure the scientific accuracy of the evaluation results.

Subsequently, based on the results of the first-level fuzzy comprehensive evaluation of the growth, yield, quality, and resource utilization indicators of *Isatis indigotica*, this study conducted a second-level weighted fuzzy comprehensive evaluation and obtained that the evaluation index of treatment T5 was the highest, indicating that the comprehensive growth effect of *Isatis indigotica* was the best under the medium-water and medium-nitrogen treatment. This is basically consistent with the research conclusion of Sheshbahreh et al. (2019) on the water-nitrogen coupling of the medicinal plant *Echinacea purpurea*. However, the evaluation model constructed in this study still has certain limitations. The weights in the second-level evaluation model are determined by the AHP, and the results inevitably have subjective experience biases. In addition, this system does not include socio-economic indicators such as economic benefits, input costs, water use benefits, and farmers’ preferences, which will also cause certain disturbances to the comprehensive evaluation results to a certain extent. Follow-up research will further optimize the model structure, expand the evaluation dimensions, and improve the evaluation system on this basis, striving to provide a more scientific and reliable theoretical basis and decision-making reference for the efficient allocation of water and fertilizer and the optimization of cultivation plans for medicinal plants.

### 4.3 Decision-making on water-nitrogen for the comprehensive growth of *Isatis indigotica*

Efficient crop cultivation is a production activity that takes into account multiple objectives such as growth, yield, quality, and resource utilization. Rational water-fertilizer regulation is to seek the best water-fertilizer inputs to achieve these multiple objectives. Chen (2025) used the PSO for multi-objective optimization of the water-nitrogen scheme for corn soybean intercropping. Fu et al. (2021) used the PSO to optimize the controller parameters of the water-fertilizer integration system, aiming to ensure the accuracy of fertilization control. This verifies the reliability and superiority of the PSO in solving crop water-fertilizer management problems. The comprehensive growth effect of *Isatis indigotica* is affected by multiple measurement indicators. Therefore, in this study, combining the multiple regression analysis method and the multi-objective optimization method of the PSO, a regression model between water-nitrogen factors and the multi-level fuzzy evaluation index was established. It was found that the influence of water-nitrogen factors on the comprehensive growth of *Isatis indigotica* showed a trend of first increasing and then decreasing, that is, there is an optimal interval. The regression model constructed by Chen et al. (2024) with the water consumption and nitrogen application rate of edible sunflower as independent variables also showed similar results. Then, the PSO was coupled for optimization, and the MATLAB software was used to analyze the model. The optimal water - regulation level was obtained as 75.10%–85.10%*θ_f_*, and the nitrogen application rate was 224.53 kg·ha^-1^, which basically corresponds to treatment T2.

## 5 Conclusion

1. The responses of various indicators of *Isatis indigotica* to water-nitrogen regulation are different. A comprehensive evaluation system was constructed based on 13 indicators of 4 types of attributes, including the growth, yield, quality, and resource utilization of *Isatis indigotica*. The fuzzy evaluation of the indicators in each factor set shows that the *YI* and *GI* of the medium water and medium nitrogen treatment (T5) were relatively the highest, with average values of 0.1173 and 0.1125, respectively. The *RUI* of the low-water or medium-water and low-nitrogen treatment (T1 or T4) was relatively high, with average values of 0.1220 and 0.1224, respectively. And the *QI* of the low-water treatment (W1) was relatively large, with an average value of 0.1025.
2. From the evaluation index of the second-level fuzzy comprehensive evaluation, the value of treatment T5 was the largest, with an average value of 0.1112; the values of treatment T4 and T6 were relatively high, with average values of 0.1052; the value of the control CK was the smallest, with an average value of. It can be seen that the medium-water and medium-nitrogen treatment can comprehensively promote multiple indicators of *Isatis indigotica* well.
3. A multiple regression model was constructed based on the coded values of water-nitrogen factors and the second-level fuzzy evaluation index. The influence of the two factors on the comprehensive growth of *Isatis indigotica* both showed a trend of first increasing and then decreasing. Then, the PSO was coupled for optimization decision-making on the water-nitrogen irrigation of *Isatis indigotica*. It was concluded that when the water-regulation level is 75.10%–85.10%*θ_f_* and the nitrogen application rate is 224.53 kg·ha^-1^, this area is conducive to achieving high-quality and high-efficiency production of *Isatis indigotica*.

In conclusion, this study took the medicinal plant *Isatis indigotica* as the research object and constructed a multi-level fuzzy comprehensive evaluation model. Judging from the evaluation results of various indicators, it is in good agreement with the measured results, overcoming the problem of the limited accuracy of a single evaluation model. At the same time, the PSO was coupled for mathematical simulation analysis of the evaluation results, improving the rationality of the water and fertilizer application amounts for *Isatis indigotica*. This can provide a methodological reference for the optimized water and fertilizer management of similar crops.

## Data Availability

The original contributions presented in the study are included in the article/supplementary material. Further inquiries can be directed to the corresponding author.
